# Proteomic Studies on the Effects of Lipo-Chitooligosaccharide and Thuricin 17 under Unstressed and Salt Stressed Conditions in *Arabidopsis thaliana*

**DOI:** 10.3389/fpls.2016.01314

**Published:** 2016-08-30

**Authors:** Sowmyalakshmi Subramanian, Alfred Souleimanov, Donald L. Smith

**Affiliations:** Department of Plant Science, McGill UniversityMontréal, QC, Canada

**Keywords:** *Arabidopsis thaliana*, Lipo-chitooligosaccharide, Thuricin17, NaCl salt stress, shotgun proteomics

## Abstract

Plants, being sessile organisms, are exposed to widely varying environmental conditions throughout their life cycle. Compatible plant-microbe interactions favor plant growth and development, and help plants deal with these environmental challenges. Microorganisms produce a diverse range of elicitor molecules to establish symbiotic relationships with the plants they associate with, in a given ecological niche. Lipo-chitooligosaccharide (LCO) and Thuricin 17 (Th17) are two such compounds shown to positively influence plant growth of both legumes and non-legumes. *Arabidopsis thaliana* responded positively to treatment with the bacterial signal compounds LCO and Th17 in the presence of salt stress (up to 250 mM NaCl). Shotgun proteomics of unstressed and 250 mM NaCl stressed *A. thaliana* rosettes (7 days post stress) in combination with the LCO and Th17 revealed many known, putative, hypothetical, and unknown proteins. Overall, carbon and energy metabolic pathways were affected under both unstressed and salt stressed conditions when treated with these signals. PEP carboxylase, Rubisco-oxygenase large subunit, pyruvate kinase, and proteins of photosystems I and II were some of the noteworthy proteins enhanced by the signals, along with other stress related proteins. These findings suggest that the proteome of *A. thaliana* rosettes is altered by the bacterial signals tested, and more so under salt stress, thereby imparting a positive effect on plant growth under high salt stress. The roles of the identified proteins are discussed here in relation to salt stress adaptation, which, when translated to field grown crops can be a crucial component and of significant importance in agriculture and global food production. The mass spectrometry proteomics data have been deposited to the ProteomeXchange with identifier PXD004742.

## Introduction

Microbes are a key component of all ecosystems on earth, playing major roles in the bio-geochemical cycles (Falkowski et al., 2008). Compounds secreted by the bacterial population of a rhizosphere are very species and environment dependent. Two bacterial signal compounds, lipo-chitooligosaccharide (LCO) from *Bradyrhizobium japonicum* strain 532C and Thuricin 17 (Th17), a bacteriocin from *Bacillus thuringiensis* strain NEB17, were isolated from bacteria that reside in the soybean rhizosphere. These signal compounds were successfully isolated and characterized, with regard to plant growth promotion, in our laboratory in 2000 and 2006, respectively (Prithiviraj et al., 2000; Gray et al., 2006a,b). These two compounds are under evaluation for their capacity to promote plant growth and development in both legumes and non-legumes under laboratory and field conditions, and are being developed as low-input components of crop production systems for deployment under Canadian climatic conditions. While LCO technology is already in the market for commercial application, with products such as Optimize, marketed by Novozymes (now a part of BASF), Th17 is under evaluation for potential commercialization.

lipo-chitooligosaccharides, also referred to as Nod factors, have been reported to positively and directly affect plant growth and development in legumes and non-legumes; as signal compounds they were first reported by Dénarié and Cullimore (1993). Nod factors have since been reported to affect plant growth in diverse plant species such as tobacco (Schmidt et al., 1993), Norway spruce and *Picea abies* (Dyachok et al., 2000, 2002; Oláh et al., 2005), soybean (Zhang and Smith, 2001; Lindsay, 2007; Wang et al., 2012; Prudent et al., 2014), canola (Schwinghamer et al., 2014), corn (Souleimanov et al., 2002a,b; Khan, 2003; Tanaka et al., 2015) and tomato (Chen et al., 2007). The LCO induced *enod* genes in non-legumes code for defense related responses, such as chitinase and PR proteins (Schultze and Kondorosi, 1996, 1998), peroxidase (Cook et al., 1995; Lindsay, 2007; Wang et al., 2012) and enzymes of phenylpropanoid pathway, such as *L*-phenylalanine ammonia-lyase (PAL; Inui et al., 1997).

*Bacillus thuringiensis* NEB17 was isolated from soybean root nodules as a putative endophytic bacterium in 1998, in our laboratory; when co-inoculated with *B. japonicum* under nitrogen free conditions, it promoted soybean growth, nodulation and grain yield (Bai et al., 2002, 2003). Subsequently, the causative agent of plant growth promotion, a bacteriocin, was isolated from *B. thuringiensis* NEB17, and is now referred to as Thuricin 17 (Gray et al., 2006b). Thuricin 17 (Th17), applied either as leaf spray or as root drench, has positive effects on soybean and corn growth. This report, from our laboratory, was the first to indicate plant growth stimulation by a bacteriocin (Lee et al., 2009). Th17 is now being tested under field conditions and DuPont Canada Crop Protection and Pioneer Canada have confirmed the stimulation of plant growth by Th17 (unpublished data).

Plants are sessile multi-cellular organisms that cope with various environmental stressors that play a major role in the growth and development of plants. Under field conditions, they face a range of challenges, the most common being soil salinity, cold temperature and drought. During the late 1990s and the early 2000s, intense gene expression and mutant studies were conducted to identify the probable signal transduction pathways; and to understand the differences and commonalities between salt, drought and cold temperature stresses. Some of the key findings are summarized herein. These three abiotic stressors are physically different and yet elicit both specific and common gene responses. With nearly every aspect of plant physiology and metabolism being affected, a very complex network of signaling pathways exist, and help plants respond to these conditions (Zhu, 2001a,b). Salt stress creates both osmotic and ionic stress in plants. The ionic stress is very distinct, associated with high sodium (Na^+^) and potassium (K^+^) deficiency, and occurs a few days after the salt stress is perceived (Munns, 2002; Xiong et al., 2002). However, the osmotic stress component is common to all three mentioned abiotic stressors, thereby converging into the induction of common sets of genes (Shinozaki and Yamaguchi-Shinozaki, 1997; Zhu, 2001a,b). Excess salt in plants results in irregularities in ion homeostasis that are controlled by the cell via various ion transporters (SOS1, 2 and 3) that restrict Na^+^ entry into the cytoplasm and regulate its accumulation in the vacuoles, and simultaneously selectively import K^+^ ions (Hasegawa et al., 2000; Zhu, 2000). *SOS1* is now known to encode for the plasma membrane localized Na^+^/H^+^ antiporter which removes Na^+^ from the cell to the outside and *SOS2* encodes for a serine/threonine protein kinase. *SOS3* encodes for a myristoylated calcium-binding protein and senses salt specific cytosolic Ca^2+^ concentration. It interacts with *SOS2* using calcium as the second messenger and targets vegetative storage protein 2 (*VSP2*) to impart salt tolerance (Gong et al., 2001), simultaneously controlling the Na^+^/H^+^ antiporter system (Qiu et al., 2002). About 5% of *Arabidopsis thaliana* genes are involved in ion regulation (Lahner et al., 2003). Differences in calcium concentration trigger protein phosphorylation cascades that provoke mitogen-activated protein-kinases, which in turn, regulate the stress response (Chinnusamy et al., 2004).

In our previous study, regarding phytohormone quantification, we observed that LCO treated *A. thaliana* rosettes had increased levels of ABA and free SA, while the Th17 treated rosettes showed increased levels of IAA and SA (Subramanian, 2014). Since ABA regulation is observed in abiotic stress tolerance and IAA regulates protein degradation using the ubiquitin proteasome pathway, which decreases the toxic effects of ROS, we wanted to assess the role of LCO and Th17 in regulation of the proteome for plant growth promotion both under optimal and salt stressed conditions.

## Materials and Methods

### Plant Material and Treatments

Seeds of *A. thaliana* Col-0 were procured from Lehle Seeds (Round Rock, TX, USA), the seeds were planted in peat pellets and the resulting plants grown in a walk-in growth chamber (Conviron Model No. PGR15, Controlled Environments Ltd, Winnipeg, MB, Canada), set at 22 ± 2°C, with a photoperiod of 16/8 h day/night cycle and 60–70% relative humidity and photosynthetic irradiance of 100–120 μmol quanta m^-1^ s^-1^.

### Extraction and Purification of Lipo-Chitooligosaccharides (LCOs) and Thuricin 17 (Th17)

The extraction and purification of LCOs was carried out and chromatography conducted for 45 min using a linear gradient of acetonitrile from 18 to 60%, as described by Souleimanov et al. (2002a). Identification of Nod factors was conducted by comparing the retention time of standard Nod factors from strain 532C (identified by mass spectrometry).

*Bacillus thuringiensis* NEB17 was cultured in King’s B medium (King et al., 1954) as previously described (Gray et al., 2006a). Th17 isolation and purification was carried out using a HPLC following the procedures of Gray et al. (2006b). The collected material was denoted as partially purified Th17, stored at 4°C and diluted to required concentrations for all the experiments.

In all the germination experiments LCO concentrations of 10^-6^ and 10^-8^ M (referred to as LCOA and LCOB, respectively, in Figures and Tables), and Th17 concentrations 10^-9^ and 10^-11^ M (referred to as THA and THB, respectively) were used, the concentrations of which were found to be the best in plant growth response studies (Prithiviraj et al., 2000; Souleimanov et al., 2002b; Lee et al., 2009).

### Petri Plate Assay for Screening for Salt Stress

Seeds of *A. thaliana* were surface sterilized in 90% alcohol for 1 min and rinsed several times with sterile water. These seeds (25 per plate) were placed on agar plates comprised of control, 10^-6^ and 10^-8^ M LCO and 10^-9^ and 10^-11^ M Th17 treatments, to score for germination. To assess salt tolerance, the seeds (25 per plate) were placed on agar plates comprising 0, 100, 150, 200, and 250 mM NaCl in combination with 10^-6^ and 10^-8^ M LCO and 10^-9^ and 10^-11^ M Th17. Control plates were comprised of only ½ MS medium with agar and the salt controls were 100, 150, 200, and 250 mM NaCl. After 48 h of stratification, the seeds were allowed to germinate and the seedlings were allowed to grow for 20 days in a growth chamber at 22 ± 2°C, with a photoperiod of 16/8 h day/night cycle and 60–70% relative humidity and photosynthetic irradiance of 100–120 μmol quanta m^-1^ s^-1^, after which the samples from the plates were assessed for visual differences in growth. Since plants in Petri plate conditions are good for screening and not for long term growth, plants were grown in trays to assess salt stress tolerance and recovery and for label free proteomic studies.

### Tray Assay for Assessing Salt Stress Recovery of *A. thaliana*

Jiffy-peat pellets (Jiffy products, Plant Products Ltd., Brampton, ON, Canada) were soaked in water to saturation and seeds of *A. thaliana* sown on them. The trays were covered and the seeds allowed to germinate. Two and half-week-old plants were subjected to 10^-6^ and 10^-8^ M LCO and 10^-9^ and 10^-11^ M Th17 treatments, followed up by fulminant salt stress at 200, 250, and 300 mM NaCl, 48 h post bacterial signal treatments. The plants were watered regularly and allowed to grow for 15 days, after which the plants were assessed for visual symptoms of salt stress and loss of turgor.

### Leaf Proteomics using Shotgun Approach

For the proteome analysis, the rosettes sampled at 24 h post bacteria signal treatments (from control, 10^-6^ M LCO and 10^-9^ M Th17) comprised the unstressed group. The remaining plants were fulminant salt stressed at 250 mM NaCl, 48 h post bacterial signal treatments. Plants from 7 days of salt stress at 250 mM NaCl in combination with 10^-6^ M LCO and 10^-9^ M Th17 treatments were sampled as the salt stressed group. 250 mM NaCl served as the salt control. The samples were flash frozen in liquid nitrogen and stored in -80°C until protein extraction. Total proteins from the samples were extracted using a protein extraction kit (Sigma–Aldrich, PE-2305, St. Louis, MO, USA).

#### Protein Extraction

In brief, the sampled (pool of three plants per replicate) rosettes were ground to a fine powder in liquid nitrogen. Approximately 100 mg of the fine powder was placed in sterile eppendorf tubes and 1 mL of ice cold methanol (Cat no. 15468-7, Sigma–Aldrich Co., St. Louis, MO, USA) was added, vortexed, incubated in -20°C for 20 min. and centrifuged (Micro12, Fisher Scientific, Denver Instrument Co., USA) at 13,000 rpm for 7 min at 4°C. The supernatant was discarded and the procedure was repeated twice more, followed by similar incubation in acetone (Cat. no. 179124, Sigma–Aldrich, Co., St. Louis, MO, USA), both steps in order to remove phenolics and secondary metabolites that might otherwise interfere with LC-MS/MS analysis. The RW2 solution was added to the samples after removing acetone, vortexed for 30 s and incubated at room temperature (22°C) for 15 min. The samples were then centrifuged at 13,000 rpm for 10 min and the supernatant carefully collected in a fresh sterile tubes. The supernatant constituted total proteins from that sample. The proteins were then diluted and quantified using the Lowry method, and samples of 10 μg in 20 μL of 1 M urea were taken to the Institut de recherches cliniques de Montréal (IRCM) for label free proteomic analysis using LC-MS/MS.

#### Protein Profiling

The total protein extracts were then digested with trypsin and subjected to LC-MS/MS using LTQ-Velos Orbitrap (Thermo Fisher, Waltham, MA, USA). Tandem mass spectra were extracted, charge state deconvoluted and deisotoped, and all MS/MS samples were analyzed using Mascot software (Matrix Science, London, UK; version 2.3.02). Mascot was set up to search the *A. thaliana* database (txid_3702, 80416 entries) assuming the digestion enzyme trypsin. Mascot was searched with a fragment ion mass tolerance of 0.60 Da and a parent ion tolerance of 15 ppm. Carbamidomethyl of cysteine was specified in Mascot as a fixed modification. Oxidation of methionine was specified in Mascot as a variable modification.

### Criteria for Protein Identification

Scaffold (version Scaffold 4.0, Proteome Software Inc., Portland, OR), was used to validate MS/MS based peptide and protein identifications. Peptide identifications were accepted if they could be established at greater than 95.0% probability, as specified by the Peptide Prophet algorithm (Keller et al., 2002). Protein identifications were accepted if they could be established at greater than 99.0% probability and contained at least two identified peptides. Protein probabilities were assigned by the Protein Prophet algorithm (Nesvizhskii et al., 2003). Proteins that contained similar peptides and could not be differentiated based on MS/MS analysis alone were grouped to satisfy the principles of parsimony.

### Data Analysis

Experiments were structured following a completely randomized design. The SAS Statistical Package 9.3 (SAS Institute Inc., Cary, NC, USA) was used, and within this the Proc Mixed procedure and Tukey’s multiple means comparison when there was significance at the 95% confidence level. Data transformation was applied when necessary to meet the criteria for analysis of variance for seed germination.

Scaffold 4.0 was used for analyzing the proteomics data for fold change and Fisher exact test of the identified proteins after subjecting the quantitative value of the spectra to the embedded normalization. The FASTA file generated was analyzed using Blast2GO-Pro V.2.6.6 (Conesa et al., 2005; Conesa and Götz, 2008; Götz et al., 2008, 2011), for the functional annotation and analysis of the protein sequences. Apart from these, Enzyme code (EC), KEGG maps and InterPro motifs were queried directly using the InterProScan web service. The mass spectrometry proteomics data have been deposited to the ProteomeXchange Consortium^1^ via the PRIDE partner repository (Vizcaino et al., 2013) with the dataset identifier PXD004742.

## Results

### *A. thaliana* Seed Germination in Petri Plates and Trays under Unstressed Conditions and in the Presence of Salt Stress Screening, When Treated with LCO and Th17

**FIGURE 1:**
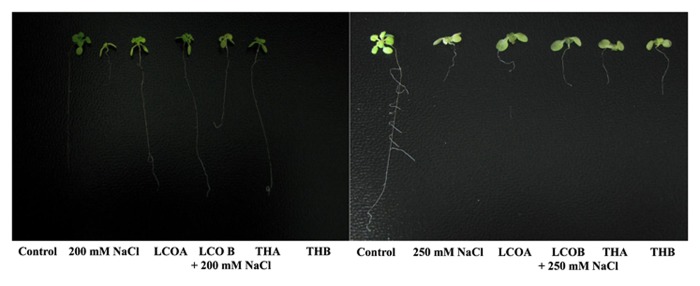
**Screening assay in petri plates for *Arabidopsis thaliana* response to 200 and 250 mM NaCl stress in the presence of lipo-chitooligosaccharide (LCO) and Th17, 20 days after imposition of salt stress.** (Control – Water; LCOA – 10^-6^ M, LCOB – 10^-8^ M, THA – 10^-9^ M, THB – 10^-11^ M; 200 and 250 mM NaCl control).

**FIGURE 2:**
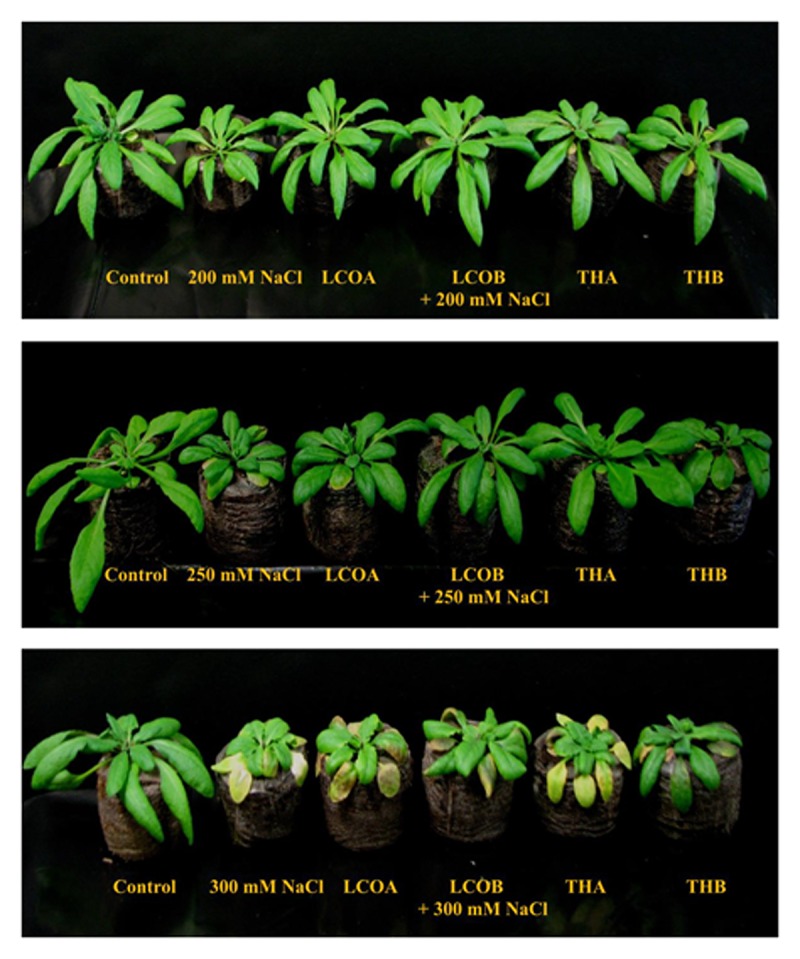
**Screening assay in trays for *A. thaliana* – visual response to different levels of salt stress in the presence of LCO and Th17, 15 days after imposition of salt stress.** (Control – Water; LCOA – 10^-6^ M, LCOB – 10^-8^ M, THA – 10^-9^ M, THB – 10^-11^ M combined with 200, 250, and 300 mM NaCl).

**FIGURE 3:**
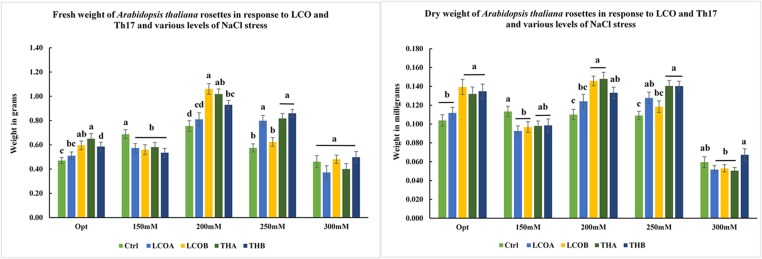
**Screening assay in trays for *A. thaliana* – fresh weight and dry weight of *A. thaliana* rosettes in response to different levels of salt stress in the presence of LCO and Th17, 15 days after imposition of salt stress.** (Optimal: Control – Water; LCOA – 10^-6^ M, LCOB – 10^-8^ M, THA – 10^-9^ M, THB – 10^-11^ M; 150, 200, 250, and 300 mM NaCl in combination with LCOA – 10^-6^ M, LCOB – 10^-8^ M, THA – 10^-9^ M, THB – 10^-11^ M treatments).

LCO and Th17 (LCO – 10^-6^ and 10^-8^ M; Th17 – 10^-9^ and 10^-11^ M) treatments generally had no effect on *A. thaliana* seed germination except at 30 h, when conditions were carefully maintained as optimal (**Table 1**). Hence an evaluation of the effects of these signal compounds in the presence of salt stress was conducted; a NaCl dosage response screening was performed. This work suggested that, in the presence of signal compounds, the plants could withstand up to 200 mM NaCl in Petri plates while the 250 mM NaCl stress completely inhibited root growth (**Figure 1**). However, the Petri plate assay is only good for early growth determinations as the seedlings are in an enclosed environment and this can induce other stresses. Hence, plants were grown in trays and two and half-week-old plants were screened with 200, 250, and 300 mM NaCl. After 48 h of treatment with LCO and Th17, the plants were treated with 200, 250, and 300 mM NaCl, allowed to recover from the shock and assessed for visual signs of stress 15 days after the NaCl stress was imposed. The plants could tolerate 250 mM NaCl while at 300 mM, the visible signs of stress were obvious such as retarded plant growth and loss of turgor (**Figure 2**). This also reflected on the plants fresh and dry weight (**Figure 3**). Hence, there were clear beneficial effects of LCO and Th17 for 250 mM NaCl treated plants and a 7 days post exposure condition was selected for rosette proteomics since the effects of salt stress are known to manifest from day 7 of exposure to NaCl salt.

**Table 1:** Least square means of *Arabidopsis thaliana* percentage germination – seeds treated with lipo-chitooligosaccharide and Thuricin 17 under optimal conditions.

Treatments	24 h	± SEM	30 h	±SEM	36 h	±SEM	48 h	±SEM
***P* ≤ 0.05**			0.1904		0.6972		0.8235	
**Control**	0.00	0.00	54.38^b^	0.97	77.49^a^	1.15	86.26^a^	1.10
**LCOA**	0.00	0.00	**61.69^ab^**	0.71	75.52^a^	1.04	87.83^a^	1.11
**LCOB**	0.00	0.00	**57.77^ab^**	0.70	72.99^a^	1.28	83.96^a^	1.57
**THA**	0.00	0.00	**62.66^a^**	0.53	78.87^a^	0.77	87.12^a^	0.76
**THB**	0.00	0.00	**61.99^ab^**	0.97	75.81^a^	1.10	86.67^a^	1.12

*Means associated with the same letter are not significantly different at p ≤ 0.05. Bold values indicate the only times when statistical significance occurred under optimal germination conditions*.

### Protein Profiling

To understand the effect of LCO and Th17 on unstressed and salt stressed *A. thailana* rosettes, total proteins were extracted from the samples and subjected to LC-MS based proteome profiling. Based on the quantitative value of the identified spectra, and at 99% protein probability, with two minimum peptides and 95% peptide probability, a number of proteins were identified in the unstressed and salt stressed treatments (**Table 2**). The treatment contrasts were then analyzed for fold-change after normalization, and Fisher’s Exact test was used to narrow down the up- and down-regulated proteins, to predict their probable functions at 24 h after signal compound treatment and 7 days after NaCl stress imposition. It is likely that we missed some of the relevant proteins due to very strict criteria for difference detection during data analysis; this level of stringency was utilized for ease of subsequent functional interpretation. According to the fold-change patterns and Fisher’s Exact test of the contrasts, the proteins were categorized as known proteins, putative proteins, hypothetical and unknown proteins (**Tables 3A**,**B**; Supplementary Datasheets 1 and 2).

**Table 2:** Total number of proteins identified at 99% protein probability and total spectra at 95% peptide probability, with two minimum peptides.

Unstressed	Control 1	Control 2	Control 3	LCO 1	LCO 2	LCO 3	Th17 1	Th17 2	Th17 3
**Proteins**	473	461	486	506	441	457	469	457	464
**Spectra**	5622	5795	5598	5910	5754	5623	5902	6009	5717
**Stressed at 250 mM NaCl**							
**Proteins**	467	491	461	456	457	506	513	514	523
**Spectra**	4753	5125	4945	4992	5098	5327	5336	5379	5626

**Table 3A:** Grouping of proteins in *A. thaliana* rosettes, that was significant in contrasts based on fold change.

Treatment contrasts *A. thaliana*	Control vs. LCO	Control vs. Th17	LCO vs. Th17	Control 250 mM NaCl vs. LCO + 250 mM NaCl	Control 250 mM NaCl vs. Th17 + 250 mM NaCl	LCO 250 mM NaCl vs. Th17 + 250 mM NaCl
**Total significant proteins**	114	93	80	93	92	80
**Known proteins**	97	80	68	72	80	64
**Putative proteins**	10	8	8	13	15	11
**Hypothetical proteins**	3	1	2	1	-	1
**Unknown proteins**	4	4	2	7	7	4

**Table 3B:** Grouping of proteins in *A. thaliana* rosettes, that were significant in contrasts based on Fisher’s Exact test.

Treatment contrasts *A. thaliana*	Control vs. LCO	Control vs. Th17	LCO vs. Th17	Control 250 mM NaCl vs. LCO + 250 mM NaCl	Control 250 mM NaCl vs. Th17 + 250 mM NaCl	LCO 250 mM NaCl vs. Th17 + 250 mM NaCl
**Total significant proteins**	63	42	26	25	40	25
**Known proteins**	55	33	22	21	34	20
**Putative Proteins**	7	8	2	4	5	5
**Hypothetical proteins**	1	1	1	-	-	-
**Unknown proteins**	-	-	1	-	1	-

Based on the known and predicted proteins, prominent proteins functioning under unstressed control conditions included *O*-methyltransferase, pyrophosphatase, COR15A and B, legume lectin family, actin 7, membrane associated progesterone binding protein, legume lectin family, a chloroplast drought induced stress protein, phosphoglycerate kinase, mitochondrial HSP70, thiamin C, profilin, TIC 40 (TRANSLOCON AT THE INNER ENVELOPE MEMBRANE OF CHLOROPLASTS) universal stress protein and a putative jasmonate inducible protein. Some of the notable proteins up-regulated in LCO treated plants were members of 40 and 60 S ribosomal protein family, phosphoenolpyruvate carboxylase (PEPC) 1 and 2, proteins of the photosystem I subunit and photosystem II 47kD, D1 and D2 proteins, acetyl CoA carboxylase, calcium sensing receptor (CaS), lipoxygenase, RUBISCO large subunit, members of oxidoreductases, fibrillin family, TIC 40 and 110, LEA protein family, peroxisomal glycolate oxidase, cadmium sensitive, cell division cycle related protein and vestitone reductase. Th17 treated rosettes had all of the above proteins up-regulated in LCO treated rosettes in addition to COR13, hydroxyl-proline rich, a major latex protein, catalase, light harvesting complex proteins LHB1B1, LHCA2, LHCB5, LHCB6, nodulin related, Zn binding oxidoreductases and progesterone binding protein (Supplementary Datasheets 1 and 2 for fold change and Fisher’s exact test results for *A. thaliana* signals group contrasts).

The number of significant proteins identified in the salt stressed signals group did not alter much in number, but did so in the types of proteins that seemed to be regulated in response to salt stress. Some of the prominent proteins of the 250 mM salt stress control were 40, 50, and 60 S ribosomal proteins, a methyl jasmonate esterase, CaS, cadmium sensitive protein 1, pyruvate decarboxylase, phosphoglycerate kinase, seed maturation protein, stromal ascorbate peroxidase, TIC 40, cinnamyl-alcohol dehydrogenase, pyruvate kinase, peroxidase, Fe-superoxide dismutase, chitinase, plastocyanin, and profiling 1. The LCO treated and salt stressed group, however, up-regulated a very different set of proteins comprised of COR15A, cytochrome B5 isoform E, glucose-phosphate-6-isomerase, LHCB 4.2 and protein D1 of photosystem II, nodulin related protein, photosystem 1 P700 chlorophyll apoprotein A1, plastid-lipid associated protein, NADH-cytochrome B5 reductase, NADPH oxidoreductases, allene oxide synthase and cyclase. Th17 treated and 250 mM NaCl stressed rosettes up-regulated proteins, some of which were common to both the salt control and LCO with salt groups. Apart from these, also affected were ATP citrate synthase, alcohol and aldehyde dehydrogenases, seed maturation protein, cinnamyl-alcohol dehydrogenase 4, cadmium sensitive, glutathione synthase transferase, PIPIB (a plasma membrane water channel protein), importin subunit, APE2 (Acclimation of leaf photosynthesis), myo-inositol, isocitrate dehydrogenase and vestitone reductase. (Supplementary Datasheets 1 and 2 for Fold change and Fisher’s exact test results for *A. thaliana* signals with stress group contrasts).

Based on Blast2GO Pro results, the enzyme code distribution for both the unstressed and salt stressed rosettes were studied. A sharp increase in some of the main enzyme classes was observed within the salt stress group, as compared to those within the unstressed group (**Table 4**). The sharp increase in oxidoreductases in LCO with stress and hydrolases in Th17 with stress could be explained through possible roles in salt stress alleviation.

**Table 4:** Enzyme code distribution in un-stressed and salt stressed groups.

Main enzyme classes	Control	LCOA	THA	250 mM NaCl Control	LCOA + 250 mM NaCl	THA + 250 mM NaCl
**Oxidoreductases**	204	191 (↓6.58%)	197 (↓3.49%)	242	248 (↑2.45%)	269 (↑10.57%)
**Transferases**	91	157 (↑53.22%)	161 (↑55.55%)	170	169 (↓0.59%)	203 (↑17.7%)
**Hydrolases**	107	129 (↑ 18.64%)	136 (↑23.87%)	153	140 (↓8.87%)	166 (↑8.15%)
**Lyases**	40	63 (↑44.66%)	73 (↑58.40%)	69	72 (↑4.25%)	76 (↑9.65%)
**Isomerases**	51	42 (↓19.35%)	36 (↓34.48%)	47	36 (↓26.50%)	40 (↓16.09%)
**Ligases**	24	52 (↑73.68%)	51 (↑72%)	60	56 (↓6.89%)	65 (↑8%)

*The % increase or decrease in the main enzyme classes within the groups (Optimal and Salt stress) is mentioned in the brackets*.

**FIGURE 4:**
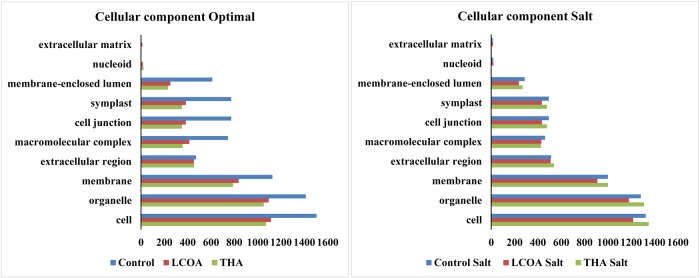
**Representation of functional classification of GO distribution for cellular components in unstressed and signals + 250 mM Salt stressed treatments in *A. thaliana* rosettes**.

**FIGURE 5:**
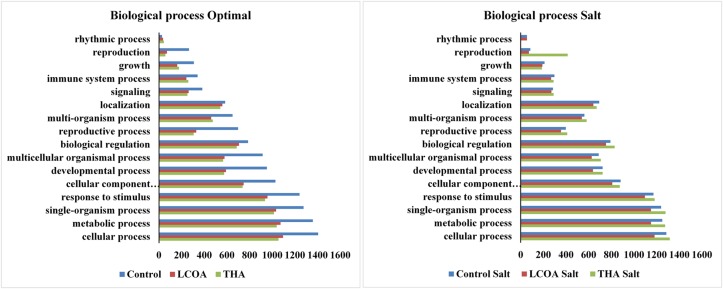
**Representation of functional classification of GO distribution for biological process in unstressed and signals + 250 mM NaCl stressed treatments in *A. thaliana* rosettes**.

**FIGURE 6:**
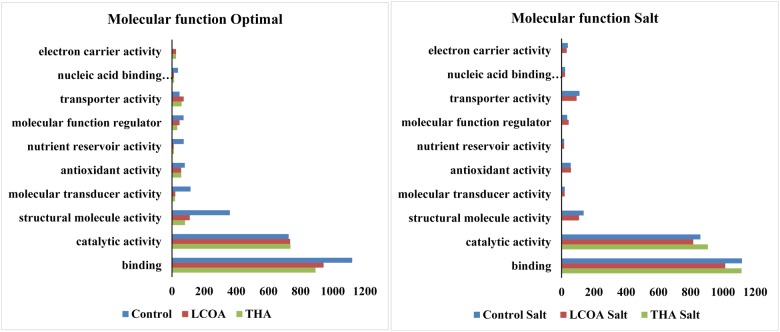
**Representation of functional classification of GO distribution for molecular function in unstressed and signals + 250 mM NaCl stressed treatments in *A. thaliana* rosettes**.

The GO function distribution characteristics of the unstressed and salt stressed groups also indicated that the proteins identified were mostly associated with ATP, GTP, protein and nucleotide binding, metal ion binding and specific to zinc, copper, cadmium, cobalt, magnesium, and calcium, response to salt and cold, glycolysis, pentose-phosphate shunt, gluconeogenesis, thylakoid associated, photorespiration, oxidation-reduction processes and photosystem II assembly (**Figures 4–6**).

Molecular function, biological processes and cellular components were all affected in both unstressed and salt-stressed conditions. Translation, translation elongation factor activity, thylakoid membrane organization proteins, starch biosynthetic process, proteins of the stromule and the extracellular region were all arrested in the salt stressed group. However, magnesium ion binding proteins, proteins related to misfolded protein responses, proteasome core complex assembly and hyperosmotic stress response, cytosolic ribosome and plant-type cell wall were all prominent in the salt stressed group. Apart from these, proteins in the cytosol, plasma membrane, chloroplast and its envelope, apoplast, plasmodesmata, nucleus and the vacuole were all up-regulated. Very little change was observed in GTP binding and GTPase activity, copper ion binding, rRNA processing, photosystem II assembly, plastoglobule, vacuole membrane, chloroplast thylakoid and stroma. The protein report for all the treatments is included as Supplementary datasheet 3 and peptide report for all the treatments is included as Supplementary datasheet 4.

## Discussion

Nod Bj V (C18:1;MeFuc), a major LCO molecule produced by *B. japonicum* 532C, isolated and identified in our laboratory, has been reported to have a positive and direct effect on both legume and non-legume seed germination, plant growth and development (Prithiviraj et al., 2003). Other than soybean and common bean, LCO can also enhance seed germination and seedling establishment in maize, rice, canola, apple, and grapes, and is accompanied by increased photosynthetic rates (Zhang and Smith, 2001). Investigations into these effects, at the molecular level, led us to transcriptomic studies, and microarray studies on soybean leaves sprayed with LCOs under optimal and sub-optimal growth conditions. The optimal condition microarray revealed 639 differentially expressed genes out of which 13 were related to abiotic stress, 14 related to biotic stress, 3 to salicylic acid and 7 to cytochrome P450s at 48 h post treatment (Lindsay, 2007). The sub-optimal stress microarray revealed the differential expression of over 600 genes. Many of these were defense and stress response related, or transcription factors suggesting the effects of LCO on the transcriptome of the leaves at 48 h post treatment (Wang et al., 2012). These results suggest a need to further explore the mechanisms by which microbe-to-plant signals might help plants accommodate abiotic and biotic stress conditions. Th17 however, has not been studied as well as LCO, as it is more recently isolated. We have some information regarding its effects on soybean and corn plant growth. The leaves of 2-week-old soybean leaves sprayed with Th17 showed increased activities of lignification-related and antioxidative enzymes and their isoforms. Both leaf spray and root drench of soybean and corn with Th17 stimulated plant growth (Jung et al., 2008; Lee et al., 2009).

Hence, in this study we subjected *A. thaliana* plants to salt stress to evaluate the efficacy of both LCO and Th17 under unstressed and salt stressed conditions for proteome profiling. *A. thaliana* is a glycophyte and sensitive to salt. The roots of *A. thaliana* seedlings were severely affected at 200 mM NaCl, in a Petri plate assay used to study salt stress (0, 50, 100, 150, 200, and 250 mM NaCl; Jiang et al., 2007). Our study shows that, in the presence of the bacterial signal compounds *A. thaliana* showed retarded root growth only at 250 mM NaCl. These compounds alleviated salt stress up to 250 mM NaCl when *A. thaliana* grown in trays were exposed to NaCl stress. At 300 mM NaCl stress, obvious stress related symptoms such as retarded plant growth and loss of turgor in the leaves were observed.

Proteins play central roles in essentially all metabolic processes. The advances in instrumentation and bioinformatic analysis have increased our understanding of proteins and their effects, which can provide key evidence regarding shifts in plant physiology. Despite these advances, proteome profiling in systems biology are still a major challenge; but the amount of information they can add to the understanding of a biological system is impressive. In this study we used the label free proteomics approach to understand *A. thaliana* proteomic responses in the presence of microbial signal compounds and under unstressed and 250 mM NaCl stressed conditions and discuss some of the key proteins identified.

Translation, translation elongation factor activity, thylakoid membrane organization proteins, starch biosynthetic process, proteins of the stromule and the extracellular region were all arrested in the salt stressed group, suggesting that the plants were arresting these processes in order to compensate for energy dependent activities associated with Na^+^ ion flushing from the cytosol. However, magnesium ion binding proteins, proteins related to cadmium response, misfolded protein responses, proteasome core complex assembly and hyperosmotic stress response, cytosolic ribosome and plant-type cell wall were all prominent in the salt stressed group. Ligands for metals such as cadmium (Cd), copper (Cu), nickel (Ni), and zinc (Zn) are seen in all plant tissues and in abundance in the xylem sap where they form complexes with histidine and citrates in the xylem sap moving from roots to leaves. The Cd binding complexes are found in both the cytosol and, predominantly, in the vacuole of the cell (Rauser, 1999). Apart from these, proteins in the cytosol, plasma membrane, chloroplast and its envelope, apoplast, plasmodesmata, nucleus and the vacuole were all up-regulated, while very little change was observed in GTP binding and GTPase activity, copper ion binding, rRNA processing, photosystem II assembly, plastoglobule, vacuole membrane, chloroplast thylakoid and stroma.

The significant findings in our study were the up-regulation of the chloroplast proteins and the proteins from photosystems I and II in the LCO and Th17 treatments, since these are generally strongly and negatively affected by salt stress. During abiotic stresses, photosynthetic capacity is reduced due to damage to photosynthetic pigments of the photosystems I and II, resulting in reduced light absorption capacity (Zhang et al., 2011; Ashraf and Harris, 2013). The stromal proteome of *Arabidopsis* chloroplasts represented 10% of the 241 proteins identified to be involved in chloroplast protein synthesis and biogenesis, with 75% being associated with the oxidative pentose phosphate pathway, glycolyis and Calvin cycle, 5–7% with nitrogen metabolism and the rest with other biosynthetic pathways such as fatty acid metabolism, amino acid metabolism, nucleotides, vitamins B1 and 2, tetrapyrroles, lipoxygenase 2 and a carbonic anhydrase (Peltier et al., 2006). The plastoglobule proteome of the chloroplast includes a M48 metallopeptidase, Absence of bc1 complex (ABC1) kinases and fibrillins, together constituting about 70 % of the plastoglobule protein biomass. The fibrillins present in other parts of the chloroplast are partitioned, probably based on their isoelectric point and hydrophobicity, to specific functions such as chlorophyll degradation and senescence, plastid proteolysis, isoprenoid biosynthesis, redox and phosphoregulation of the electron flow, although most of the functions of the associated proteins are still not clear (Lundquist et al., 2012).

Plants are exposed to various levels of light in nature and one of the ways they compensate for this is by regulating their thylakoid membrane proteins. Light harvesting protein complex protein phosphorylation is catalyzed by light-dependent protein kinase mediated by plastoquinone and is driven by the electron transport system based on light dosage (Ranjeva and Boudet, 1987). Photosystem II light interception is mediated by pigment proteins that belong to a large class of antenna pigments. Light harvesting complex II (LHC II) is the most abundant of the photosystem II proteins; the apoprotein and pigment-protein holocomplex is structurally very heterogenous. The LHC II apoproteins Lhcb1, Lhcb2, and Lhcb3 are coded by *Lhcb1, Lhcb2*, and *Lhcb3* genes. Once synthesized in the cytoplasm as precursors, these are transported into the chloroplast by post-translational modifications (Jackowski et al., 2001). A short term post-translational redistribution of LHC and a long term chloroplast DNA transcription, balance the regulation of photosystems I and II, and is dictated by the redox state of plastoquinone which is in turn controlled by chloroplast sensor kinase (CSK; Allen et al., 2011).

Exposure to light stress causes oxidative and nitrosative stress and the proteins of photosystem I and II are affected differentially. Amino acid oxidation products are determined mostly in the photosystem II reaction center, and this often leads to tyrosine and tryptophan oxidation or nitration (Galetskiy et al., 2011). About 80–200 proteins present in the thylakoid lumen are closely associated with the light harvesting complexes and the other proteins regulating photosynthesis. Following an 8 h light exposure, PsbP and PsbQ subunits of photosystem II were seen to increase along with a major plastocyanin and various proteins of unknown function. These proteins also seem to be similarly expressed at the transcription level (Granlund et al., 2009). The excess light energy perceived by plants is channeled into the chloroplasts and dispersed by the mitochondrial respiratory chain. The type II NAD(P)H dehydrogenases in the inner membrane of the mitochondria, cyanide-resistant alternative oxidase and phosphorylating pathway complexes I, III, and IV regulate this energy processing. Along with the glycine decarboxylase complex (GDC), these pathways regulate the energy balance between chloroplast and mitochondria under stressful conditions, wherein these pathways are up-regulated to maximize photosynthetic efficiency (Noguchi and Yoshida, 2008).

Carbonylation of proteins in organisms is an irreversible and oxidative process that increases with age and leads to disfunction of modified proteins in the system. In *Arabidopsis*, protein carbonylation is found to increase as the plant grows, but is seen to decrease drastically during the onset of bolting and flowering. Hsp70, ATP synthases, RUBISCO large subunit and proteins of the light harvesting complex and of energy transfer are all targets of this mechanism (Johansson et al., 2004). Despite the high salt stress levels imposed on the plants in this experiment, the photosystem proteins were still up-regulated in the LCO and Th17 treatments, suggesting that these two signals are preventing the damage of these photosystem proteins in a way we still do not understand.

The other up-regulated proteins in this experiment include those of PEPC and phosphoenolpyruvate carboxylase kinase (PPCK) in the LCO and Th17 treatments, which are two major cytosolic enzymes central to plant metabolism. During phosphate deprivation, *A. thaliana* responds by phosphorylating PEPC subunits to modulate the metabolic adaptations of lower phosphate availability (Gregory et al., 2009). The plant type – PEPC genes encode for 110-kDa polypeptides. These peptides are homotetrameric and contained several conserved sites for serine-phosphorylation and lysine-mono-ubiquitination (O’Leary et al., 2011; and references therein).

The up-regulation of the proteasome pathway indicates that the stress generated toxic proteins might be degraded by this system. In previous studies, the 20S proteasome pathway was seen to be up-regulated in both RNA and protein levels of cadmium stressed *Arabidopsis* leaves, suggesting that this proteasome pathway might help with degrading stress generated oxidized proteins (Polge et al., 2009). Also, the 26 S proteasome of *A. thaliana* contains 26 unique proteins with at least 13 of them containing tryptophan residues as identified using nano-flow liquid chromatography (Russell et al., 2013). Post-transcriptional gene regulation is, in part, controlled by RNA-binding proteins (RBP). The *A. thaliana* genome encodes for more than 200 RBPs and they contribute to diverse developmental processes, chromatin modification and environmental adaptation (Lorkovic, 2009).

LEA proteins, initially discovered and researched in seeds, are now reported to be present in other vegetative tissues and have a wide range of sequence diversity and intercellular localization, with expression patterns depending on environmental conditions. The majority of the predicted LEA proteins are highly hydrophilic and found mostly in unfolded conditions, being involved largely with cellular dehydration tolerance. In *Arabidopsis*, nine distinct groups of LEA proteins, encoded by 51 different LEA protein genes, have been reported; most harbor abscisic acid response (ABRE) and/or low temperature response (LTRE) elements in their promoters (Hundertmark and Hincha, 2008). Up-regulation of LEA proteins in LCO and Th17 under salt stress correlates with these findings in this abiotic stress tolerance mechanism.

Up-regulation of *A. thaliana* membrane-associated progesterone binding protein was observed in Th17 treatment. Progesterone 1 was detected in apple seeds as early as 1968, but due to technical challenges in instrumentation and reliability of assays, the role of progesterone in plants was conclusively established by Saden-Krehula et al. (1991; Janeczko and Skoczowski, 2005). It has now been detected in a variety of dicots and monocots such as adzuki bean, mung bean, pea, tomato, potato, apple, onion, rice, and *Arabidopsis*, with the shoots having relatively more abundant progesterone 1 than inflorescences, seeds, roots, and tubers. Progesterone 1 was also seen to promote plant growth at very low concentrations (range of 0.01–1 μM) suggesting that this could be playing the role of a hormone in plants, regulating growth and development (Iino et al., 2007; Nakano and Yokota, 2007). It is possible that this membrane associated protein functions in a way better than LCO in promoting plant growth.

Nodulin genes once thought to be specialized genes present only in legumes have been observed in some non-legumes. The role of nodulin genes might be diverse and related to general organogenesis, rather than restricted to nodulation. Nodulin genes are found in high transcript levels in floral tissues (Szczyglowski and Amyot, 2003). Other enod40 genes have been cloned from non-legumes include tomato (Vleghels et al., 2003), maize (Compaan et al., 2003), rye grass (*Lolium*) and barley (*Hordeum*) (Knud, 2003). Enod40 levels are elevated during arbuscular mycorrhizal root colonization of tobacco (*Nicotiana bentana*) and alfalfa (*Medicago truncatula*) (Sinvany et al., 2002). *Azorhizobium caulinodans* ORS571 colonized the roots of *A. thaliana* through lateral root cracks and the colonization was improved upon addition of flavonoids naringenin and daidzein. Both colonization and flavonoid stimulation were *Nod gene* independent (Gough et al., 1997). Early nodulin like protein has been observed to accumulate during the early stages of sieve cell differentiation (Khan et al., 2007). Investigation of transgenic lines of *Arabidopsis* for early nodulin gene enod40 function showed reduction in cell size of selected tissues in the plant, such as the leaf mesophyll and the epidermal internode cells (Guzzo et al., 2005). Nodulin protein analogs in watermelon control fruit development and ripening (Wechter et al., 2008). Roles for nodulin genes have been reported in tomato fruit development and ripening (Lemaire-Chamley et al., 2005). Up-regulation of nodulin related proteins in both LCO and Th17 with NaCl stress suggests that this nodulin related protein is mostly playing a role in plant development during stress tolerance.

## Conclusion

In this study, we compared the effects of LCO and Th17 under unstressed and salt-stressed conditions; this is the first study conducted to determine the effects of these signals, in combination with stressful levels of salt, on *A. thaliana* proteome. *A. thaliana* is a glycophyte and is sensitive to salt stress. LCO is commercially available (products such as Optimize with LCO promoter technology) and is known to accelerate plant growth in the field. The comparison between LCO and Th17 and the effects on the proteome of the rosettes under stressed and unstressed conditions is another step to understanding the effects of these compounds, at the proteome level, during plant growth. This study also increases our understanding of plant–microbe interactions, mainly in the use of such growth promoting technologies, boosting the potential for decreased use of synthetic chemical inputs on cultivated land, and perhaps enhanced crop productivity on salinized soils around the world.

## Author Contributions

SS designed, performed experiments and data analysis; AS and DS contributed to reagents/materials/analysis tools; SS and DS wrote the manuscript.

## Conflict of Interest Statement

The authors declare that the research was conducted in the absence of any commercial or financial relationships that could be construed as a potential conflict of interest.

## Footnotes

1 http://proteomecentral.proteomexchange.org
